# Comonomer Reactivity Trends in Catalytic Ethene/1-Alkene Copolymerizations to Linear Low-Density Polyethylene

**DOI:** 10.3390/polym17172290

**Published:** 2025-08-24

**Authors:** Gianluigi Galasso, Roberta Cipullo, Vincenzo Busico, Antonio Vittoria

**Affiliations:** Department of Chemical Sciences, Federico II University of Naples, via Cinthia, 80126 Napoli, Italy; gianl.galasso@studenti.unina.it (G.G.); rcipullo@unina.it (R.C.)

**Keywords:** Linear Low-Density Polyethylene, ethene copolymerization, higher 1-alkenes, molecular catalysts, Ziegler–Natta catalysts, reactivity ratios

## Abstract

Linear Low-Density Polyethylene (LLDPE) is a versatile polyolefin made by copolymerizing ethene with minor amounts of a 1-alkene. The short side chain branches in the comonomer units partly hinder the ability of the polyethylene main chain to crystallize, thus providing a way to fine-tune material properties between the extremes of a thermoplastic and a moderate elastomer. In this function, higher 1-alkenes such as 1-hexene or 1-octene are more effective than shorter homologs like propene or 1-butene, because their alkyl substituents are fully incompatible with the polyethylene lattice. On the other hand, the former comonomers are also more expensive and, above all, poorly reactive with heterogeneous Ziegler–Natta (ZN) catalysts, the workhorses of the polyolefin industry; as a matter of fact, they can only be used with technologically more demanding molecular catalysts. The molecular kinetic factors governing this important and complicated catalytic reactivity are still poorly understood, and perusal of the literature led us to conclude that data reliability is often questionable due to experimental limitations in reaction equipment and protocols, particularly in academic laboratories. In this study, we made use of a state-of-the-art High-Throughput Experimentation workflow to measure the reactivity ratios with ethene of two representative higher 1-alkenes, namely 1-hexene and 1-decene, in the presence of a variety of well-defined molecular catalysts of metallocene and post-metallocene nature comparatively with a typical MgCl_2_/TiCl_4_ ZN catalyst for polyethylene application. We found that the two comonomers react almost identically with molecular catalysts, whereas a major decrease in reactivity for 1-decene compared with 1-hexene was observed idiosyncratically for the ZN catalyst. In our opinion, the overall results suggest that in the latter case, surface effects can be dominant over direct comonomer interactions with the coordination sphere of the active metal in dictating the observed molecular kinetic behavior.

## 1. Introduction

Linear Low-Density Polyethylene (LLDPE) is a rapidly growing polyolefin produced by copolymerizing ethene with a higher 1-alkene (e.g., 1-butene, 1-hexene, 1-octene) in the presence of a coordination catalyst [1,2]. The short side chain branches introduced along the polyethylene (PE) main chain partly hinder crystallization, and materials ranging from thermoplastics to plastomers and moderate elastomers can be obtained by choosing the proper 1-alkene comonomer identity and relative amount [3,4,5,6,7]. The ability of the comonomer to disrupt PE crystallinity increases with the increasing size of the alkyl substituent on the olefinic bond, but the cost grows in the same direction, so that 1-hexene and 1-octene represent good compromises [6,8,9].

**Figure 1:**
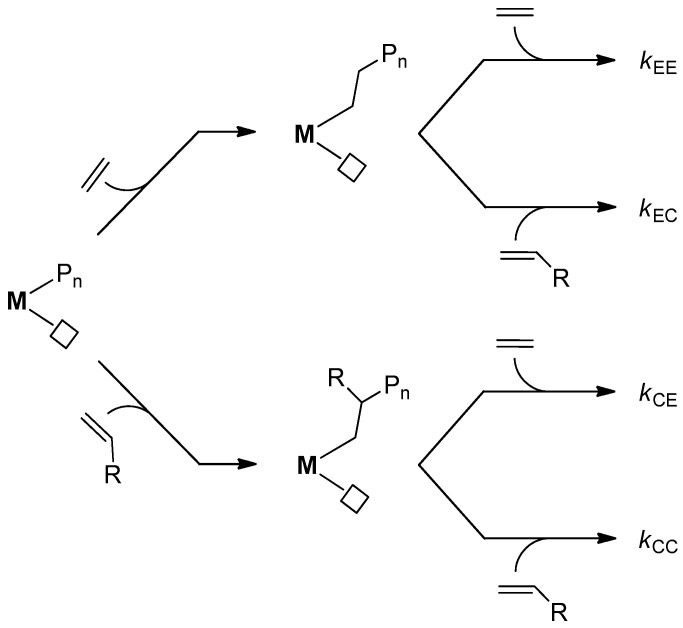
Schematic representation of ethene/1-alkene copolymerization assuming first-order Markov statistics. M, transition metal; R, alkyl; P_n_, growing polymer chain; □, coordination vacancy; E, ethene; C, 1-alkene comonomer; *k*_EE_, *k*_EC_, *k*_CE_, *k*_CC_, apparent kinetic constants of monomer insertion for the four elementary steps.

The copolymerization process is well-described, assuming first-order Markov statistics and the four elementary steps depicted in Figure 1 [10,11,12].

The following relationships can be derived from the general copolymerization equation [13]:

$$P_{\mathrm{EE}}\;=\;1\;-{\;P}_{\mathrm{EC}}\;=\;\frac{\mathrm{EE}}{\;E}$$

$$P_{\mathrm{CC}}\;=\;1\;-\;P_{\mathrm{CE}}\;=\;\frac{\mathrm{CC}}{C}$$

$$r_{E}\;=\;\frac{k_{\mathrm{EE}}}{k_{\mathrm{EC}}}=\;\frac{P_{\mathrm{EE}}}{{\;P}_{\mathrm{EC}}}\;\cdot \;\frac{\left[C\right]}{\left[E\right]}\;=\;\frac{P_{\mathrm{EE}}}{1\;-{\;P}_{\mathrm{EE}}}\;\cdot \;\frac{\left[C\right]}{\left[E\right]}$$

$$r_{C}\;=\;\frac{k_{\mathrm{CC}}}{k_{\mathrm{CE}}}=\;\frac{P_{\mathrm{CC}}}{P_{\mathrm{CE}}}\;\cdot \;\frac{\left[E\right]}{\left[C\right]}\;=\;\frac{P_{\mathrm{CC}}}{1\;-{\;P}_{\mathrm{CC}}}\;\cdot \;\frac{\left[E\right]}{\left[C\right]}$$

where *P*_XY_ is the probability of inserting monomer Y after monomer X (X and Y = E or C), and E_n_ (C_n_) is the fractional abundance of an E (C) homosequence of length n in the copolymer.

Heterogeneous MgCl_2_/TiCl_4_ Ziegler–Natta (ZN) [14,15,16,17] and Cr-based Phillips type [18,19,20,21,22] catalysts, which dominate the industrial production of the ethene homopolymer (High-Density Polyethylene, HDPE) for an unbeatable cost-to-performance balance, are very poor (Phillips) or poor (ZN) 1-alkene incorporators due to very high *r*_E_ and very low *r*_C_ values. ZN LLDPE grades are produced commercially with the ethene/1-butene (E/B) comonomer pair, but copolymer composition distribution is broad and multimodal, spanning from a B-poor fraction that resembles HDPE to a weakly crystalline or even amorphous B-rich fraction [23,24,25]. This undesired feature becomes more prominent with the increasing size of the 1-alkene, to the point that comonomers like 1-hexene or 1-octene do not represent viable options [26,27,28]. Therefore, molecular catalysts, which do not suffer from said limitations, are progressively gaining ground notwithstanding a much higher cost [14,29,30,31,32,33,34,35,36].

The mechanistic origin (s) of the above facts is (are) cloudy. The presence of multiple active species with largely different reactivities is a common occurrence in heterogeneous catalysts and is likely the reason for the multimodal composition distribution of ZN LLDPE materials [37,38,39,40]. On the other hand, the severe drop of reactivity of higher 1-alkenes with ZN active species is more difficult to understand, due to the ill-defined structure of the active TiCl_n_ species and of their surface environment on the nanocrystalline MgCl_2_ support. Comparative studies with molecular catalysts can be very useful in this respect, because Quantitative Structure–Property Relationships (QSPRs) are much easier to determine when the chemical structure of the (pre) catalysts is known and can be fine-tuned through the proper choice and design of the ancillary ligands [41,42,43,44,45,46,47,48]. Somewhat surprisingly, though, the systematic literature studies of ethene copolymerizations with higher 1-alkenes in the presence of metallocene or ‘post-metallocene’ catalysts are rather limited in number and partly controversial in their conclusions [3,31,49,50,51,52,53,54]. Precisely and accurately dosing gaseous monomers and ensuring a constant comonomer feeding ratio in batch or semi-batch ethene copolymerization processes with highly active catalysts are technically demanding operations, and we suspect that the seemingly conflicting results in the literature can be traced at least in part to inadequate reaction execution and control.

In the present work, we applied a state-of-the-art High-Throughput Experimentation (HTE) workflow to screen a library of diverse molecular catalysts in semi-batch copolymerizations of ethene with 1-hexene and 1-decene, two representative liquid 1-alkenes, aiming to find out if and to what extent an increase in the size of the alkyl substituent on the double bond leads to a decrease in relative reactivity in various well-defined catalytic pockets. Monomer reactivity ratios were measured by means of ^13^C NMR comonomer sequence analyses on the copolymers. The results were then compared with homologous ones for a typical ZN catalyst used under similar conditions, to highlight analogies or differences between the various catalyst classes. For reasons that will become clear in the following sections, solvent (diluent) effects were explicitly explored and were found to be relevant in the context of interest.

## 2. Materials and Methods

**Figure 2:**
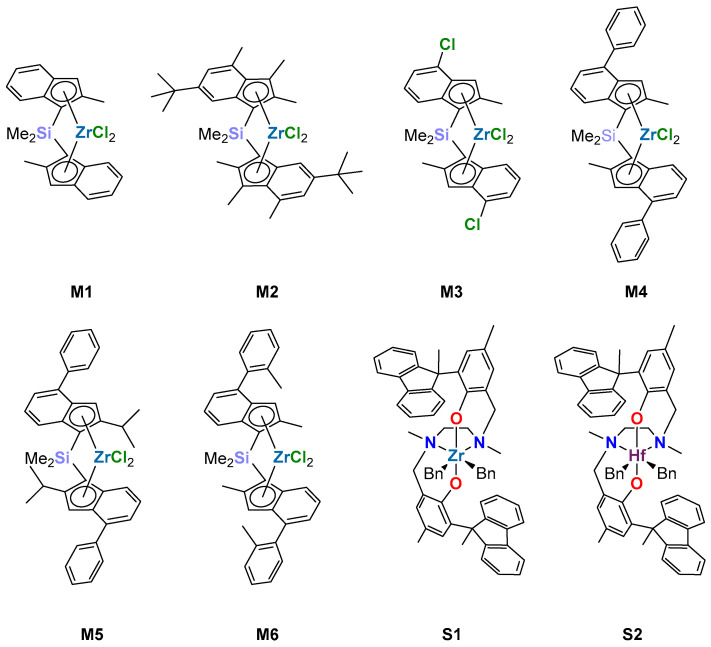
Chemical structures of the molecular precatalysts used in this work.

Figure 2 shows the structures of the eight molecular precatalysts selected for this study. These feature different coordination geometries (pseudo-tetrahedral for *ansa*-zirconocenes **M1–M6**, pseudo-octahedral for the two Salan ‘post-metallocenes’ **S1–S2**), two Group 4 metals (Zr and Hf), and various ligand substitution patterns, ensuring widely diverse steric and electronic properties. The synthesis of precatalysts **M2**, **M3**, **M5**, **M6**, **S1** and **S2** was described before [41,55,56]. Precatalysts **M1** and **M4** and a sample of MgCl_2_/TiCl_4_ ZN precatalysts for PE application (**ZN-1**, Ti content = 11.9 wt.%) were kindly donated by SABIC Europe.

The copolymerization reactions were performed in a Freeslate (former Symyx, Santa Clara, CA, USA) Parallel Pressure Reactor with 48 reaction cells (PPR48), integrally contained in a triple MBraun (Munich, Germany) glovebox under a nitrogen atmosphere. The reaction cells of the PPR48 feature magnetically coupled mechanical stirring (up to 800 rpm). Gases are fed into the cells by way of solenoid valves, whereas liquids and slurries are delivered robotically with dedicated injection systems. Each cell is electronically controlled and monitored in real time for temperature (±0.1 °C), pressure (±2 psi), gaseous monomer uptake, and uptake rate. We have successfully applied this setup in many studies of homogeneous and heterogeneous catalytic olefin polymerizations [57,58,59,60,61].

In the present investigation, all ethene/1-alkene copolymerization experiments were carried out in semi-batch operation mode. The liquid 1-alkene was pre-loaded in excess, and ethene was fed on demand at a proper partial pressure until a desired conversion was attained, at which point the reaction was quenched with an overpressure of dry air. The Design of Experiments (DoE) is provided in the Supplementary Materials, while the copolymerization protocol is described below.

Prior to loading the reaction cells, the PPR modules underwent ‘Bake and Purge’ cycles overnight (i.e., an intermittent flow of dry N_2_ at 90–140 °C over 8 h, to remove any impurities left from previous experiments). Afterwards, the modules were cooled down to glovebox temperature, the stir tops were removed, and the reaction cells were fitted with disposable glass vials containing 5.2 mL of a liquid solution made of solvent (diluent), 1-alkene comonomer, and scavenger, pre-dispensed manually by the operator utilizing single-channel pipettes.

Disposable PEEK paddles were then fitted to the stirrers, and the stir tops were placed back to close the modules. A pressure test with N_2_ at 80 or 150 psi, depending on the DoE, was carried out to check that all modules were pressure-tight, after which N_2_ was replaced with ethene gas at 15 psi, the modules were thermostated at the copolymerization temperature (60 °C), an overpressure of 15 psi of a 20/80 H_2_/N_2_ mixture was added if required by the DoE, and finally the modules were pressurized with further ethene gas at the working pressure (65 or 130 psi, depending on the DoE). After reaching saturation of the reaction phases with ethene, the catalyst injection sequence was started: 0.8 mL of liquid consisting of a first aliquot of solvent (diluent) used as ‘chaser’, a solution of the precatalyst, a solution of the activator, and a second aliquot of solvent (diluent) used as a ‘buffer’, all separated by nitrogen gaps to prevent mixing and undesired premature precatalyst activation, were robotically uploaded in the reported order into the PPR injection syringe and subsequently injected into each cell through a pressure-tight rubber septum, thus starting the copolymerization reaction. The reactions were allowed to proceed under vigorous stirring (800 rpm) until the desired conversion of ethene was reached and then quenched by admitting an over-pressure of dry air.

After quenching all cells, the modules were cooled down to glovebox temperature and vented. The stir tops were removed, and the glass vials with the reaction phases were taken out and transferred to a Martin Christ (Osterode, Germany) RVC 2–33 CDplus centrifugal evaporator where the produced copolymers were dried overnight. Reaction yields were measured by weighing the glass vials with the dry copolymers with a Mettler-Toledo (Columbus, OH, USA) Bohdan Balance Automator and subtracting the pre-recorded tare. The copolymer samples were finally submitted to the characterizations illustrated below.

For the copolymers made with molecular catalysts, Gel Permeation Chromatography (GPC) curves were recorded with a Freeslate (Sunnyvale, CA, USA) Rapid GPC setup equipped with a set of 2 mixed-bed Agilent (Santa Clara, CA, USA) PLgel 10 μm columns and a Polymer Char (Valencia, Spain) IR4 detector. The upper deck of the setup accommodates a 48-well polymer dissolution station with magnetically stirred 10 mL glass vials. With robotic operation, pre-weighed polymer amounts (2–3 mg) were dissolved in proper volumes of 1,2-dichlorobenzene (ODCB) containing 0.40 mg mL^−1^ of butylated hydroxytoluene (BHT) stabilizer, so as to obtain solutions at a concentration of 0.5–1.0 mg mL^−1^. The polymer solutions were maintained at 150 °C under gentle stirring for 2 h, subsequently transferred to a thermostated bay at 145 °C, and injected sequentially into the column line at 145 °C and a flow rate of 1.0 mL min^−1^. In post-trigger delay operation mode, the analysis time was 12.5 min per sample. The instrument was calibrated with the universal method, using 10 monodisperse polystyrene samples (*M*_n_ between 1.3 and 3700 kDa). Before and after each campaign, samples from a known ethene/1-octene copolymer benchmark batch were analyzed for a consistency check.

For the copolymers made with the ZN catalyst, GPC curves were collected with a Polymer Char GPC-IR setup equipped with an autosampler (42 wells) and an IR6 detector. With robotic operation, pre-weighed amounts of polymer (typically, 6.0 mg) were dissolved in ODCB added with 0.40 mg·mL^−1^ of BHT stabilizer, to achieve 0.75 mg·mL^−1^ solutions. After 90 min at 145 °C under vortexing in sealed vials to ensure complete dissolution, the samples were sequentially charged into the columns through an injection loop. The instrument was calibrated with the universal method, using 16 monodisperse polystyrene samples (*M*_w_ between 0.266 and 12,900 kDa). Before and after each campaign, samples from a known ethene/1-octene copolymer benchmark batch were analyzed for a consistency check.

The copolymer samples made with the ZN catalyst were also characterized by means of analytical Crystallization Elution Fractionation (CEF) [62]. The CEF traces were collected with a Polymer Char CEF setup equipped with an autosampler (42 wells), an IR5 detector, and a dual-capillary viscometer detector. With robotic operation, pre-weighed amounts of copolymer (typically, 6.0 mg) were dissolved in ODCB added with 0.40 mg mL^−1^ of BHT stabilizer, to achieve a concentration of 0.75 mg mL^−1^. After 90 min at 150 °C under vortexing in sealed vials to ensure complete dissolution, the samples were sequentially charged into the injection loop, where they were held at 95 °C for 5 min and then moved to the column. The crystallization step entailed a cooling ramp down to 35 °C at a rate of 2 °C min^−1^ and a flow of 0.05 mL min^−1^. Then, 1 min after reaching 35 °C, sample elution was started, with a heating ramp up to 140 °C at a rate of 4 °C min^−1^ and a flow of 1.0 mL min^−1^. The analysis time was 60 min per sample.

^1^H and ^13^C NMR spectra of all copolymer samples were recorded with a Bruker (Billerica, MA, USA) Avance III 400 spectrometer (400 MHz for ^1^H) equipped with a high-temperature cryoprobe for 5 mm OD tubes and an autosampler with 24 pre-heated positions. The samples (∼25 mg) were dissolved at 120 °C in 1,1,2,2-tetrachloroethane-*d*_2_ (∼0.6 mL) and loaded in the carousel maintained at the same temperature. The spectra were acquired sequentially with automated tuning, matching, and shimming. Operating conditions were as follows: [^1^H-NMR] 90° pulse; 2.0 s acquisition time; 10 s relaxation delay; 16–32 transients; [^13^C-NMR] 45° pulse; 2.3 s acquisition time; 2.0 s relaxation delay for homopolymers; 5.0 s relaxation delay for copolymers; 1 K to 3 K transients; and broad-band proton decoupling with a modified WALTZ16 sequence (BI_-WALTZ16_32 by Bruker). Comonomer sequence distributions at triad level were determined from quantitative ^13^C NMR spectra according to methods in the literature [13,52,63,64].

Full operating details for all the characterizations can be found in the Supplementary Materials.

## 3. Results and Discussion

A library of eight *C*_2_-symmetric molecular precatalysts (chemical structures in Figure 2) was selected for this study. The set includes six pseudo-tetrahedral bis (1-Indenyl) *ansa*-zirconocenes and two octahedral Salan Group 4 ‘post-metallocenes’ featuring different metals (Zr and Hf). The resulting catalytic pockets have large steric and electronic diversity [65,66,67]; as a matter of fact, the values of monomer reactivity ratios previously measured in several investigations of ethene/1-alkene copolymerizations [12,30,42] span a very wide range (*r*_E_ values between 2 and 190).

A first set of semi-batch ethene/1-hexene (E/H) and ethene/1-decene (E/D) copolymerization experiments were carried out at 60 °C, with the aim of preparing copolymers with accurately controlled copolymer compositions enabling reliable measurements of ^13^C NMR comonomer sequence distributions (*vide infra*). Using the protocol already described in the previous section, the PPR reaction cells were loaded with proper amounts of toluene, liquid 1-alkene comonomer, and triisobutyl-Al (TIBA) as alkylating agent and scavenger, the reaction phases were saturated with ethene (fed from the gas phase at a desired partial pressure), and the reactions were started by injecting toluene solutions of the precatalysts and a tetrakis-perfluorophenylborate activator. Comonomer feeding ratios were kept nearly constant by continuously feeding ethene on demand under vigorous mechanical stirring and quenching the reactions with dry air at ‘low’ (<15%) 1-alkene conversion. To the best of our knowledge, this protocol represents the most widely used and effective approximation of the behavior of a continuous copolymerization reactor [42,68,69].

Table 1 summarizes the results for each of the eight catalysts. These include (a) copolymer composition (*x*_C_) at the set value of the comonomer feeding ratio, measured by ^13^C NMR; (b) the two monomer reactivity ratios (*r*_E_ = *k*_EE_/*k*_EC_, *r*_C_ = *k*_CC_/*k*_CE_, and C = Comonomer), determined by statistical analysis of the ^13^C NMR comonomer sequence distribution at triad level, assuming first-order Markov statistics [12] as explained in the Materials and Methods section; and (c) the values of *M*_n_ and *M*_w_/*M*_n_, measured by high-temperature GPC. The *M*_w_/*M*_n_ ratio, in particular, is a valuable parameter for judging whether or not a copolymerization reaction is adequately controlled [70,71,72,73]: based on previous experience with the PPR48 setup, a value of *M*_w_/*M*_n_ < 2.3 is close enough to the theoretical one for a Schulz–Flory distribution (*M*_w_/*M*_n_ = 2.0, expected for a single-center catalytic process) to rule out the hypothesis of cell over-heating or the excessive drift of the comonomer feeding ratio.

**Figure 3:**
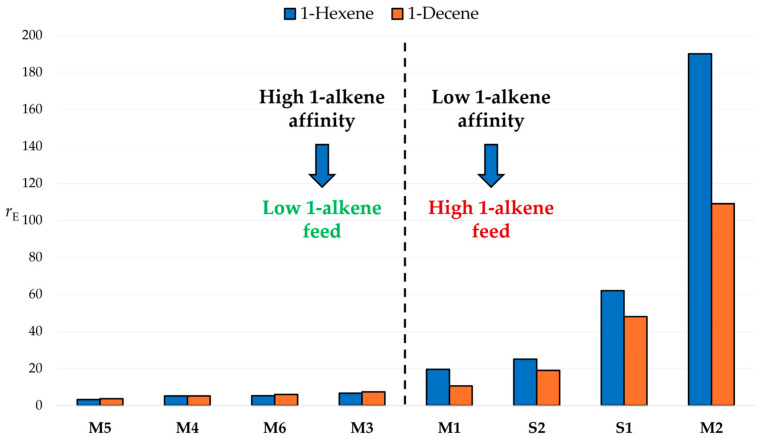
Values of *r*_E_ measured by ^13^C NMR analysis of the triad distributions in the copolymers of Table 1 (see text; catalyst ID codes are after Figure 2).

Not surprisingly, the monomer reactivity ratios *r*_E_ and *r*_C_ were strongly dependent on catalyst structure; however, for each given catalyst, they turned out to be quite similar (within a factor 2) for the two ethene/1-alkene comonomer pairs. A slightly higher reactivity of 1-hexene relative to 1-decene was observed for four out of the eight catalysts in the library; on the other hand, quite unexpectedly, the opposite was found for the other four catalysts, namely, **M1**, **M2**, **S1** and **S2**, which rather favored 1-decene. Notably, the catalysts belonging in the latter subset all featured high values of *r*_E_, which forced us to operate at high 1-alkene concentrations in the liquid phase to reach the desired comonomer content in the produced copolymer (Table 1 and Figure 3); our educated guess was that the solvation properties of the reaction medium were substantially affected by this condition.

**Figure 4:**
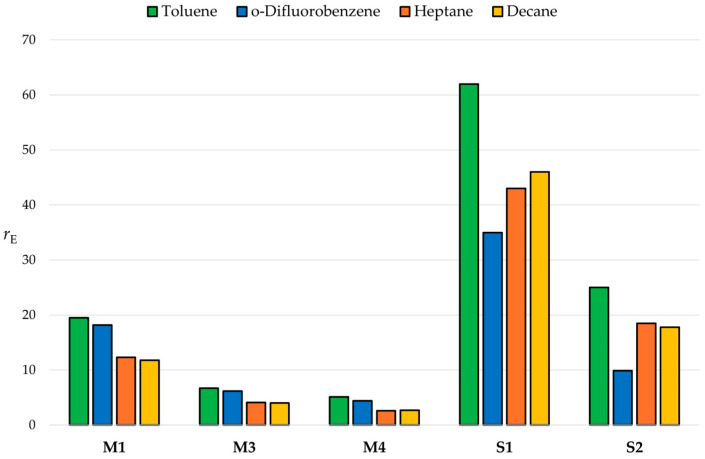
Values of *r*_E_ measured by ^13^C NMR analysis of the comonomer triad distribution in the copolymers of Table 1 and Table 2 (see text; catalyst ID codes are after Figure 2).

It is worth recalling that all eight catalysts in active form are ion pairs, and the possible occurrence of solvent effects modulating relative comonomer reactivities is not unlikely [34,74,75,76]; that this can be the case was shown by, e.g., Forlini et al. for propene/1-hexene copolymerizations [77]. To explore this hypothesis, ethene/1-hexene copolymerization experiments like those in Table 1 were repeated for a subset of five catalysts spanning a range of *r*_E_ values between 5.1 and 62 in three different solvents, namely, heptane, decane and 1,2-difluorobenzene. The main results are shown in Table 2 and Figure 4.

Consistently with the hypothesis, for all catalysts in the subset, *r*_E_ turned out to be sensitive to the choice of solvent, by up to a factor 2 and in a complex manner: in fact, for the three *ansa*-zirconocenes, *r*_E_ was higher in aromatic than in aliphatic solvents, whereas for the two Salan post-metallocenes, *r*_E_ was highest in toluene and lowest in 1,2-difluorobenzene. It should be noted that the measured concentration of ethene after saturation of the liquid phase was substantially independent of the solvent (Table S6 in the Supplementary Materials). Whatever the mechanistic origin (s) of these observations, we conclude that differences in the nature and composition of the reaction medium cannot be disregarded when comparatively evaluating monomer reactivity ratios for different comonomer pairs; apart from the cited ref. [77], we have not found any mention to this fact in the previous literature.

In view of this, we carried out a third set of ethene/1-alkene copolymerization experiments at 60 °C in toluene at low 1-alkene concentrations, so to operate practically in the same reaction medium (>96 vol% toluene) for all catalysts and with both comonomers. The results are shown in Table 3.

Average catalyst productivities at a low comonomer concentration were generally higher compared to the experiments in Table 1, and a tendency toward copolymer precipitation was observed in at least some cases; the slight broadening of some *M*_w_/*M*_n_ values in Table 3 can be traced to these two facts and a consequent modest deterioration of reaction control. Comonomer incorporations in the copolymers produced were too low for accurate measurements of monomer reactivity ratios from the ^13^C NMR triad distributions. However, all experiments were carried out at the same comonomer feeding ratio, and therefore direct comparisons of copolymer composition (*x*_C_ values) for each given catalyst and comonomer pair were feasible [33]. Notably and importantly, in all cases, the two homologous E/H and E/D copolymers turned out to have identical compositions within the experimental uncertainty, which led us to conclude that the relative reactivities of 1-hexene and 1-decene are the same, independently of individual catalyst structures and monomer reactivity ratios. The approximate values of *r*_E_ in the last column of Table 3 are consistent with such a conclusion.

This is evidently at odds with the behavior of ZN catalysts as reported in the literature [26,27,28] and recalled in the Introduction. To achieve an independent confirmation of such an idiosyncratic behavior, we carried out comparative ethene/1-hexene and ethene/1-decene copolymerization experiments in the PPR in the presence of a MgCl_2_/TiCl_4_ ZN catalyst for PE application (**ZN-1**) under experimental conditions identical to Table 1, except for the cocatalyst and scavenger (triethyl-Al instead of TIBA) and the hydrocarbon diluent (heptane instead of toluene). The results, reported in Table 4, confirmed that the catalyst is a very poor 1-hexene incorporator (*on average*, similarly to zirconocene **M2**) and point out a further drop in comonomer reactivity with 1-decene by roughly one order of magnitude, which seems unique to this catalyst class.

For all copolymers, GPC-IR and CEF characterizations (Figures S2–S5 in the Supplementary Materials) highlighted the expected microstructural dis-uniformity. Indeed, the samples were separated by CEF into a higher-molar-mass HDPE-like fraction and a lower-molar-mass fraction with rather high comonomer content, to the point that the latter fraction was eluted at room temperature. Whether this should be attributed to inherently different structures of the active TiCl_n_ adsorbates on the MgCl_2_ support [38,39,40,78] and/or to diverse surface locations of otherwise similar adsorbates on the MgCl_2_ crystallites (e.g., plain surfaces vs. edges or corners) is presently unknown.

## 4. Conclusions

In this study, we comparatively investigated ethene/1-hexene and ethene/1-decene copolymerizations mediated by a variety of molecular (metallocene and post-metallocene) catalysts and one representative heterogeneous MgCl_2_/TiCl_4_ ZN catalyst. Based on the results of systematic copolymer synthesis and characterization experiments carried out in a High-Throughput Experimentation workflow under highly controlled and truly comparable reaction conditions, we concluded that the inherent reactivity of 1-hexene and 1-decene with molecular catalysts in liquid phase is practically identical; this holds irrespectively of whether the catalyst is a good or poor 1-alkene incorporator. On the other hand, for the ZN catalyst, we found that, *on average*, 1-decene was roughly 10-fold less prone than 1-hexene to be incorporated into the copolymer chains, which was very peculiar. Generally accepted models of ZN active sites feature an octahedral coordination environment of the active Ti [79,80,81] resembling that of Zr and Hf in the two post-metallocenes **S1** and **S2** (Figure 2). This can justify very high values of *r*_E_ in ethene/1-alkene copolymerizations, but it does not explain the observed drop in relative reactivity when changing the comonomer from 1-hexene to 1-decene. Based on the results presented in this study, we suggest that MgCl_2_ surface topology should be considered among the structural elements that can dictate copolymerization behavior: 1-alkene molecules with long conformationally flexible alkyl substituents on the double bond may well undergo *specific* and *strong* excluded volume effects and adsorption interactions with the surface, and the exact location of a catalytic species should be enormously important in that respect. Comparing copolymer composition distributions in the ethene/1-alkene copolymerization products of crystalline (and therefore inherently anisotropic) or amorphous (and as such relatively isotropic) heterogeneous catalyst systems can shed light on this point and will be the subject of forthcoming investigations.

## Figures and Tables

**Table 1:** Results of ethene/1-hexene and ethene/1-decene copolymerizations at 60 °C in toluene for the catalysts of Figure 2 (see text and Supplementary Materials, Table S2).

Catalyst	Comonomer	*V*_C_ μL	[C] mol/L	*R*_P_ kg_pol_mmol_Cat_^−1^h^−1^	*x*_C_ mol%	*r*_E_	*r*_C_	*M*_n_ kDa	*M*_w_/*M*_n_
**M1**	1-hexene ^#^	3400	4.5	26	34.1	19.5	0.011	32	2.2
	1-decene	5100	4.5	46	46.2	10.5	0.028	26	2.1
**M2**	1-hexene ^#^	4250	5.7	11	8.4	190	0.002	105	2.2
	1-decene	5100	4.5	20	11.4	109	0.004	90	2.2
**M3**	1-hexene ^#^	850	1.1	9.1	38.4	6.7	0.22	52	2.1
	1-decene	1300	1.1	6.6	35.7	7.3	0.20	53	2.1
**M4**	1-hexene ^#^	850	1.1	13	40.7	5.1	0.19	85	2.1
	1-decene	1300	1.1	9.4	40.5	5.2	0.18	88	2.3
**M5**	1-hexene ^#^	850	1.1	0.42	48.0	3.2	0.20	18	2.3
	1-decene	1300	1.1	0.73	50.8	3.7	0.19	25	2.2
**M6**	1-hexene ^#^	850	1.1	5.3	45.9	5.3	0.29	76	2.2
	1-decene	1300	1.1	5.6	41.3	6.0	0.25	100	2.3
**S1**	1-hexene	2125	2.8	28	23.0	62	0.14	35	2.1
	1-decene	5100	4.5	25	49.6	48	0.18	25	2.1
**S2**	1-hexene	2125	2.8	30	40.6	25	0.16	31	2.2
	1-decene	3200	2.8	35	47.9	19.0	0.18	35	2.0

*p*_E_ = 65 psi. *V*_C_ = volume of liquid 1-alkene in the reaction cell. [C] = 1-alkene comonomer concentration in the reaction phase. *R*_p_ = average catalyst productivity. *x*_C_ = comonomer fraction in the copolymer. ^#^ data from reference [42], obtained using an identical experimental setup and copolymerization protocol and conditions.

**Table 2:** Results of ethene/1-hexene copolymerization at 60 °C in three different solvents for a subset of five catalysts from Figure 2 (see text and Supplementary Materials, Table S3; DFB, 1,2-difluorobenzene).

Catalyst	Solvent	*V*_H_ μL	*R*_P_ kg_pol_mmol_Cat_^−1^h^−1^	*x*_H_ mol%	*r*_E_	*r*_H_	*M*_n_ kDa	*M*_w_/*M*_n_
**M1**	DFB	3400	36	30.6	18.2	0.035	49	2.1
	heptane		50	41.3	12.3	0.025	26	2.2
	decane		46	43.7	11.8	0.024	27	2.1
**M3**	DFB	850	9.3	38.9	6.2	0.23	45	2.1
	heptane		18	47.8	4.1	0.34	43	2.1
	decane		18	52.5	4.0	0.34	39	2.2
**M4**	DFB	850	3.6	41.2	4.4	0.18	90	2.4
	heptane		26	53.3	2.6	0.36	89	2.1
	decane		33	55.8	2.7	0.33	77	2.1
**S1**	DFB	2125	5.1	35.4	34	0.17	26	2.0
	heptane		8.8	29.7	43	0.16	27	2.1
	decane		7.6	33.1	46	0.16	29	2.0
**S2**	DFB	2125	4.0	57.7	9.8	0.22	28	2.0
	heptane		2.0	47.2	18.3	0.22	27	2.1
	decane		1.4	52.4	17.5	0.21	25	2.0

*p*_E_ = 65 psi. *V*_H_ = volume of liquid 1-hexene in the reaction cell. *R*_p_ = average catalyst productivity. *x*_H_ = hexene comonomer fraction in the copolymer.

**Table 3:** Results of ethene/1-hexene and ethene/1-decene copolymerizations at 60 °C in toluene at low 1-alkene concentrations (see text and Supplementary Materials, Table S4).

Catalyst	Comonomer	*V*_C_ μL	*R*_P_ kg_pol_mmol_Cat_^−1^h^−1^	*x*_C_ mol%	*M*_n_ kDa	*M*_w_/*M*_n_	*r*_E_ ^#^
**M1**	1-hexene	150	108	1.3	57	2.0	25
	1-decene	225	69	1.4	55	2.1	23
**M2**	1-hexene	150	63	0.35	129	2.3	94
	1-decene	225	149	0.35	136	2.2	93
**M3**	1-hexene	150	16	4.3	62	2.0	7.2
	1-decene	225	13	3.8	59	2.2	8.2
**M4**	1-hexene	150	30	4.5	73	2.5	6.9
	1-decene	225	45	4.7	79	2.4	6.5
**M5**	1-hexene	150	190	7.9	190	2.6	3.7
	1-decene	225	176	8.3	176	2.6	3.4
**M6**	1-hexene	150	4.1	4.2	112	2.1	7.5
	1-decene	225	12	4.0	111	2.2	7.0
**S1**	1-hexene	150	325	0.51	105	2.4	64
	1-decene	225	74	0.56	104	2.4	58
**S2**	1-hexene	150	166	1.5	166	3.2	21
	1-decene	225	191	1.8	191	3.8	18

*p*_E_ = 130 psi. *V*_C_ = volume of liquid 1-alkene in the reaction cell. *R*_p_ = average catalyst productivity. *x*_C_ = comonomer fraction in the copolymer. ^#^ Indicative value estimated as *r*_E_ ∼ [C]·(1 − *x*_C_)/([E]·*x*_C_).

**Table 4:** Results of ethene/1-hexene and ethene/1-decene copolymerizations at 60 °C in the presence of ZN catalyst **ZN-1** in heptane slurry (see text and Supplementary Materials, Table S5).

Catalyst	Comonomer	*V*_C_ μL	*x*_C_ mol%	*M*_n_ kDa	*M*_w_/*M*_n_	*r*_E_ ^#^
**ZN-1**	1-hexene	3370	6.7	55	8.5	2.1 × 10^2^
	1-decene	5100	1.0	119	6.1	1.5 × 10^3^

*p*_E_ = 65 psi, *p*_H2_ = 3.0 psi. *V*_C_ = volume of liquid 1-alkene in the reaction cell. *x*_C_ = comonomer fraction in the copolymer. ^#^ Indicative value estimated as *r*_E_ ∼ [C]·(1 − *x*_C_)/([E]·*x*_C_).

## Data Availability

The original contributions presented in the study are included in the article and the Supplementary Materials; further inquiries can be directed to the corresponding author.

## Supplementary Materials

The following supporting information can be downloaded at https://www.mdpi.com/article/10.3390/polym17172290/s1, Figure S1: Typical pressure profile of a reaction cell during a copolymerization experiment; Table S1: List of chemicals employed in this work; Table S2: Details of individual copolymerization experiments in Table 1 (main text) and results of copolymer characterizations (see Section 2 and main text); Table S3: Details of individual copolymerization experiments in Table 2 (main text) and results of copolymer characterizations (see Section 2 and main text; DFB = 1,2-difluorobenzene); Table S4: Details of individual copolymerization experiments in Table 3 (main text) and results of copolymer characterizations (see Section 2 and main text); Table S5: Details of individual copolymerization experiments in Table 4 (main text) and results of copolymer characterizations (see Section 2 and main text); Figure S2: CEF trace of copolymer sample #1 of Table S5 (soluble fraction SF = 47 wt%); Figure S3: CEF trace of copolymer sample #4 of Table S5 (soluble fraction SF = 8.3 wt%); Figure S4: GPC/IR trace of copolymer sample #1 of Table S5; Figure S5: GPC/IR trace of copolymer sample #4 of Table S5; Table S6: Results of saturation experiments of pure solvents (toluene, 1,2-difluorobenzene, *n*-heptane and decane) with ethene at 60 °C in the PPR. The authors have cited additional references [82] in the Supplementary Materials.

## Abbreviations

The following abbreviations are used in this manuscript:

LLDPE	Linear low-density polyethylene
PE	Polyethylene
ZN	Ziegler–Natta
HDPE	High-density polyethylene
QSPR	Quantitative Structure–Property Relationship
HTE	High-Throughput Experimentation
PPR	Parallel Pressure Reactor
DoE	Design of Experiment
GPC	Gel Permeation Chromatography
ODCB	1,2-Dichlorobenzene
BHT	Butylated Hydroxytoluene
CEF	Crystallization Elution Fractionation
TIBA	Triisobutyl-aluminum
