# Supplementary material for: Comonomer Reactivity Trends in Catalytic Ethene/1-Alkene Copolymerizations to Linear Low-Density Polyethylene
Source: Polymers (Basel). 2025 Aug 24;17(17):2290. doi: 10.3390/polym17172290 (PMC12431525; doi:10.3390/polym17172290)
Supplement: Supplementary file 1 [file polymers-17-02290-s001.zip › polymers-3822886-supplementary.pdf]

Supplementary Materials

Comonomer reactivity trends in catalytic ethene/1-alkene copolymerizations to Linear Low-Density Polyethylene

Gianluigi Galasso[1], Roberta Cipullo[1], Vincenzo Busico[1*] and Antonio Vittoria[1*]

[1] Department of Chemical Sciences, Università di Napoli Federico II, Via Cintia, 80126 Napoli, Italy;

*E-mail: busico@unina.it; antonio.vittoria@unina.it

Table of Contents

1. General remarks and list of chemicals 3

2. Ethene/1-alkene copolymerization and copolymer characterization protocols 3

3. Copolymer synthesis and characterization details 5

4. Liquid saturation experiments with ethene 10

5. References 11

1. General remarks and list of chemicals

Air-sensitive compounds were manipulated under inert atmosphere (N_2 or Ar) inside MBraun LabMaster gloveboxes. Materials used in the glovebox to store/handle air-sensitive compounds were treated for at least 8 hours in vacuum ovens. A list of suppliers and specifications of the chemicals used in this work is reported in **Table S1**.

Table S1. List of chemicals employed in this work.

Chemical	Supplier	Notes
Decane	Carlo Erba	Purified in a MBRAUN SPS-5 unit by passing in sequence through fixed-bed columns filled with (a) A4 molecular sieves and (b) activated-Cu
n-Heptane	Carlo Erba	
1,2-difluorobenzene (DFB)	ROMIL	
Toluene	Carlo Erba	
Isododecane	SABIC Europe	Pure, dry
Ethene	Linde	3.5 grade; purified by passing through a mixed-bed activated-Cu/A4 molecular sieves column
H_2/N_2 mixture	SIAD	5.0 grade
1-Hexene	SABIC Europe	Purified by passing in sequence through a 13X molecular sieves and an activated-Cu fixed-bed column
1-Decene	SABIC Europe	
Anilinium Tetrakis(pentafluorophenyl) Borate (AB)	TCI	>98% pure
Triphenylmethyl Tetrakis(pentafluorophenyl) Borate (TTB)	Acros Organics	97% pure
Triethylaluminium (TEA)	Chemtura Europe	Neat, ≥ 95.5%
Tri-*iso*-butylaluminium (TIBA)	Sigma Aldrich	Neat, ≥ 97%
1,2-dichlorobenzene (ODCB)	Carlo Erba	>99.8% isomeric purity
1,1,2,2-Tetrachloroetane-d_2	ARMAR Chemicals	99.5% isotopic purity

2. Ethene/1-alkene copolymerization and copolymer characterization protocols

All copolymerization experiments were performed in a Freeslate (former Symyx) Parallel Pressure Reactor (PPR) platform with 48 reaction cells arrayed in six 8-cell modules, integrally contained in a triple MBraun LabMaster glovebox under nitrogen.

PPR reaction cells feature magnetically coupled mechanical stirring (800 rpm), and are individually controlled in real time with respect to temperature, pressure, monomer gas uptake and uptake rate. Gases (e.g. nitrogen, ethene, hydrogen, dry air) are fed into the cells by way of solenoid valves. Ethene/1-alkene copolymerization reactions were carried out in semi-batch mode, pre-loading an excess of liquid 1-alkene

and feeding ethene on demand at a proper partial pressure until a desired conversion was attained, at which point the reaction was quenched with an excess of dry air. The pressure of each cell was constantly monitored by the PPR operating system and kept at the setpoint within a ±2.0 psi deadband by properly operating the solenoid valves. A typical pressure profile is shown in **Figure S1**.

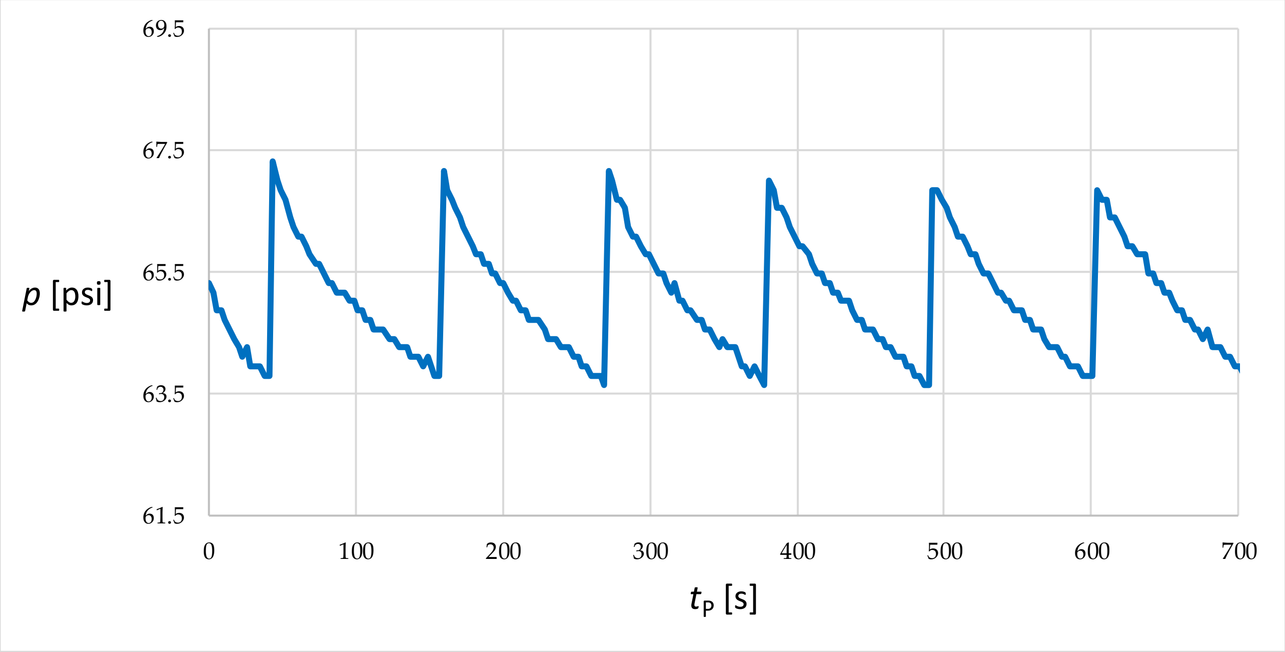

Figure S1. Typical pressure profile of a reaction cell during a copolymerization experiment.

Liquid or slurries can be injected into the PPR cells by means of a robotic arm operating a syringe with a specially designed stainless steel injection needle. After loading the syringe with the desired volumes of liquids or slurries from glass or stainless steel vials kept under vortexing, the arm moves the needle to the injection port of each cell, where the needle pierces a pressure-tight rubber septum, penetrates the gas cap and dispenses its contents directly to the liquid phase, thus ensuring highly accurate and precise dosing. The injections are executed according to a Design of Experiment (DoE) prepared in advance by the operator using a dedicated software (Library Studio); however, DoE modifications are possible during the execution of a library of experiments. The reaction phases are contained in glass vials fitted in advance in the cells, for easy withdrawal, workup and cleaning. The general reaction protocol has been described before, and successfully applied in several olefin polymerization studies.[1–5]

The copolymers were characterized by Gel Permeation Chromatography (GPC) for the determination of molecular weight distributions, Crystallization Elution Fractionation (CEF) and ^{13}C NMR spectroscopy for the determination of comonomer sequence distribution. Statistical analysis of the ^{13}C NMR comonomer sequence data was carried out according to literature and assuming a 1st-order Markov statistics. [6–9] Ethene concentration was calculated from saturation experiments (see **Section 4**). For 1-alkene concentration, an average value between the initial and final value was considered and calculated as follows:

$$[C]_i = \frac{n_{C,\,i}}{V_{tot}}$$

$$[C]_f = \frac{n_{C,\,f}}{V_{tot}} = \frac{n_{C,\,i} - \dfrac{\text{Yield} \cdot x_C(\text{wt\%})}{MW_C}}{V_{tot}}$$

3. Copolymer synthesis and characterization details

Copolymerization experiments with molecular catalysts

Solutions of precatalysts and activators were prepared by dissolving weighted amounts of the solids in a known volume of the reaction solvent, dispensed using tared pipettes. Al-alkyl solutions were prepared by diluting aliquots of the neat compound in the reaction solvent.

5 mmol of TIBA were added to each PPR reaction cell as scavenger. Catalysts **M1-6** were activated using AB as activator with [AB]/[M] = 2.0 (M = transition metal). Catalysts **S1** and **S2** were activated using TTB as activator with [TTB]/[M] = 0.9. The details of individual copolymerization experiments and the results of copolymer characterizations are reported in **Tables S2-S4**.

Table S2. Details of individual copolymerization experiments in **Table 1** (main text) and results of copolymer characterizations (see **Section 2** and main text).

Cat.	1-alkene	V_C (μL)	n_{cat} (nmol)	Yield (mg)	t_P (s)	R_P ($kg_{pol}mmol_{Cat}^{-1}h^{-1}$)	M_n (kDa)	M_w (kDa)	$[E]/[C]_{av}$	$[C]_{conv}$ (%)	x_C (mol%)	P_{EE}	P_{CC}
M1	1-hexene*	3400	20	106	739	26	32	69	0.0672	2.8	34.1	0.567	0.140
	1-decene	5100	15	147.5	1027	34	26	55	0.0679	3.2	45.7	0.412	0.287
	1-decene	5100	15	165.5	680	58	26	54	0.0680	3.6	46.7	0.423	0.297
M2	1-hexene*	4250	15	57.5	1292	11	105	231	0.0531	0.4	8.4	0.910	0.039
	1-decene	5100	10	44.1	831	19	92	205	0.0670	0.5	11.4	0.882	0.059
	1-decene	5100	15	57.4	690	20	89	199	0.0670	0.6	11.5	0.877	0.054
M3	1-hexene*	850	20	104	2036	9.1	52	108	0.281	11.8	38.4	0.655	0.443
	1-decene	1300	20	90.7	2362	6.9	54	113	0.272	7.0	37.0	0.659	0.441
	1-decene	1300	20	94.9	2670	6.4	52	112	0.272	7.1	34.4	0.670	0.412
M4	1-hexene*	850	30	97.0	845	13	85	182	0.281	11.4	40.7	0.589	0.397
	1-decene	1300	20	102.7	2378	7.8	85	205	0.273	8.2	39.8	0.593	0.393
	1-decene	1300	20	109.5	1837	11	91	198	0.274	8.8	41.1	0.585	0.407
M5	1-hexene*	850	40	42.0	9001	0.42	18	41	0.272	5.4	48.0	0.463	0.421
	1-decene	1300	60	117.7	10451	0.68	25	56	0.276	10.2	50.5	0.506	0.399
	1-decene	1300	60	144.2	11085	0.78	24	54	0.280	12.6	51.1	0.507	0.405
M6	1-hexene*	850	20	54.0	1819	5.3	76	166	0.2741	6.8	45.9	0.594	0.517
	1-decene	1300	30	108.2	2112	6.1	93	226	0.2741	8.8	41.5	0.619	0.478
	1-decene	1300	30	109.5	2515	5.2	106	238	0.2742	8.8	41.2	0.624	0.477
S1	1-hexene	2125	30	80.0	330	29	35	74	0.107	2.6	22.6	0.870	0.552
	1-hexene	2125	30	80.0	342	28	35	72	0.107	2.6	23.4	0.870	0.564
	1-decene	5100	30	142.4	847	26	26	54	0.0679	3.1	49.4	0.755	0.729
	1-decene	5100	30	145.0	814	24	24	51	0.0679	3.2	49.9	0.772	0.730
S2	1-hexene	2125	300	126.0	701	27	27	58	0.109	5.9	40.7	0.727	0.596
	1-hexene	2125	30	105.0	6518	34	34	74	0.109	4.9	40.4	0.731	0.595
	1-decene	3200	300	135.9	560	32	32	65	0.109	4.8	49.6	0.675	0.622
	1-decene	3200	300	140.7	634	38	38	78	0.109	4.8	46.2	0.674	0.614

*Data from reference [1], obtained using an identical experimental setup and copolymerization protocol and conditions.

Table S3. Details of individual copolymerization experiments in **Table 2** (main text) and results of copolymer characterizations (see **Section 2** and main text; DFB = 1,2-difluorobenzene).

Cat.	Solvent	V_H (μL)	n_{cat} (nmol)	Yield (mg)	t_P (s)	R_P ($kg_{pol}mmol_{Cat}^{-1}h^{-1}$)	M_n (kDa)	M_w (kDa)	$[E]/[H]_{av}$	$[H]_{conv}$ (%)	x_H (mol%)	P_{EE}	P_{HH}
M1	DFB	3400	8	94.2	2742	15	50	106	0.0688	2.3	30.3	0.564	0.334
	DFB	3400	15	140.2	603	56	47	102	0.0692	3.5	31.0	0.550	0.336
	n-heptane	3400	10	128.1	916	50	26	59	0.0708	3.8	41.3	0.463	0.250
	n-heptane	3400	10	131.6	927	51	27	58	0.0708	3.9	41.3	0.467	0.265
	decane	3400	10	131.2	1043	45	27	56	0.0646	4.0	43.4	0.444	0.276
	decane	3400	10	131.9	1029	46	27	57	0.0647	4.0	44.0	0.419	0.271
M3	DFB	850	10	67.5	3677	6.6	47	96	0.290	7.9	40.5	0.633	0.455
	DFB	850	10	70.2	2097	12	44	94	0.290	7.9	37.3	0.654	0.431
	n-heptane	850	10	84.7	2273	13	43	91	0.307	10.8	47.7	0.556	0.520
	n-heptane	850	10	103.6	1654	23	44	92	0.311	13.3	47.8	0.556	0.529
	decane	850	10	98.7	1840	19	38	83	0.259	13.3	52.6	0.506	0.566
	decane	850	10	100.9	2007	18	39	85	0.260	13.6	52.5	0.514	0.564
M4	DFB	850	20	72.9	5401	2.4	94	215	0.292	8.7	41.5	0.558	0.383
	DFB	850	20	96.2	3601	4.8	85	208	0.296	11.4	41.0	0.565	0.383
	n-heptane	850	15	128.1	1320	23	89	189	0.318	17.3	53.2	0.442	0.527
	n-heptane	850	15	128.8	1121	28	90	186	0.318	17.4	53.3	0.456	0.530
	decane	850	15	141.1	1129	30	76	165	0.269	19.8	57.3	0.403	0.560
	decane	850	15	146.2	979	36	78	164	0.269	19.9	54.2	0.436	0.544
S1	DFB	2125	30	107.3	3368	3.8	28	55	0.113	4.6	35.5	0.789	0.606
	DFB	2125	30	113.6	2128	6.4	23	49	0.113	4.9	35.3	0.797	0.602
	n-heptane	2125	30	83.3	1048	9.5	28	58	0.116	3.1	28.7	0.838	0.582
	n-heptane	2125	30	83.7	1249	8.0	26	55	0.116	3.3	30.7	0.825	0.595
	decane	2125	30	72.4	1179	7.4	30	60	0.101	3.0	33.0	0.824	0.617
	decane	2125	30	85.1	1288	7.9	28	57	0.101	3.5	33.2	0.821	0.618
S2	DFB	2125	200	194.5	814	4.3	29	56	0.116	10.7	56.3	0.544	0.641
	DFB	2125	200	202.4	948	3.8	26	53	0.117	11.4	59.1	0.521	0.658
	n-heptane	2125	200	143.8	1225	2.1	27	58	0.118	7.2	46.1	0.680	0.648
	n-heptane	2125	200	143.9	1316	2.0	28	59	0.118	7.3	48.2	0.687	0.655
	decane	2125	200	145.1	1975	1.3	25	53	0.103	7.6	51.6	0.653	0.669
	decane	2125	200	163.5	2006	1.5	24	49	0.104	8.7	53.2	0.636	0.680

Table S4. Details of individual copolymerization experiments in **Table 3** (main text) and results of copolymer characterizations (see **Section 2** and main text).

Cat.	1-alkene	V_C (µL)	n_{cat} (nmol)	Yield (mg)	t_P (s)	R_P ($kg_{pol}mmol_{Cat}^{-1}h^{-1}$)	M_n (kDa)	M_w (kDa)	[E]/[C]$_{av}$	[C]$_{conv}$ (%)	x_C (mol%)
M1	1-hexene	150	5	61.9	411	108	57	112	3.04	2.3	1.3
	1-decene	225	5	47.6	499	69	55	114	3.06	1.9	1.4
M2	1-hexene	150	5	50.7	582	63	129	299	3.01	0.5	0.35
	1-decene	225	7.5	123.7	399	149	136	293	3.05	1.3	0.35
M3	1-hexene	150	5	38.7	1739	16	62	125	3.07	4.6	4.3
	1-decene	225	7.5	42.9	1647	13	59	133	3.09	4.2	3.8
M4	1-hexene	150	3	39.1	1567	30	73	182	3.08	4.8	4.5
	1-decene	225	3	49.5	1331	45	79	189	3.12	5.9	4.7
M5	1-hexene	150	40	44.3	3601	190	190	500	3.14	9.0	7.9
	1-decene	225	40	63.9	3631	176	176	459	3.22	11.9	8.3
M6	1-hexene	150	7.5	28.6	3331	4.1	112	240	3.05	3.3	4.2
	1-decene	225	10	54.5	1697	12	111	247	3.12	5.6	4.0
S1	1-hexene	150	2.5	90.9	403	325	105	254	3.02	1.4	0.51
	1-decene	225	2	44.7	1084	74	104	254	3.04	0.7	0.56
S2	1-hexene	150	35	72.9	342	166	166	1020	3.05	3.2	1.5
	1-decene	225	40	75.2	310	191	191	1036	3.09	3.8	1.8

Copolymerization experiments with the ZN catalyst system

Precatalyst slurries were prepared by suspending a weighted amount of solid in isododecane. Solutions of TEA were prepared as described in the previous section. All reactions were carried out at 60°C. 15 psi of a 20/80 H_2/N_2 (p_{H2} = 3 psi) gas were added to the reaction cells as a chain transfer agent, and 1.0 µmol of TEA as a scavenger. The **ZN-1** precatalyst was activated with TEA at [Al]/[Ti] = 100. Details on the single polymerization experiments are reported in **Table S5**.

Table S5. Details of individual copolymerization experiments in **Table 4** (main text) and results of copolymer characterizations (see **Section 2** and main text).

Experiment/ Sample #	1-alkene	V_C (µL)	m_{cat} (mg)	n_{Ti} (nmol)	Yield (mg)	t_P (s)	R_P ($kg_{pol}mmol_{Ti}^{-1}h^{-1}$)	M_n (kDa)	M_w (kDa)	[E]/[C]$_{av}$	[C]$_{conv}$ (%)	x_C (mol%)
1	1-hexene	3370	0.015	37	38.7	6219	0.61	58	520	0.0669	0.3	5.7
2			0.030	75	55.0	634	4.2	53	416	0.0670	0.5	7.7
3	1-decene	5100	0.0075	19	44.5	650	14	120	740	0.0668	0.1	0.9
4			0.0075	19	45.8	640	14	118	711	0.0668	0.1	1.1

CEF and GPC/IR traces for the copolymer samples #1 and #4 of **Table S5** are shown in **Figures S2-5**.

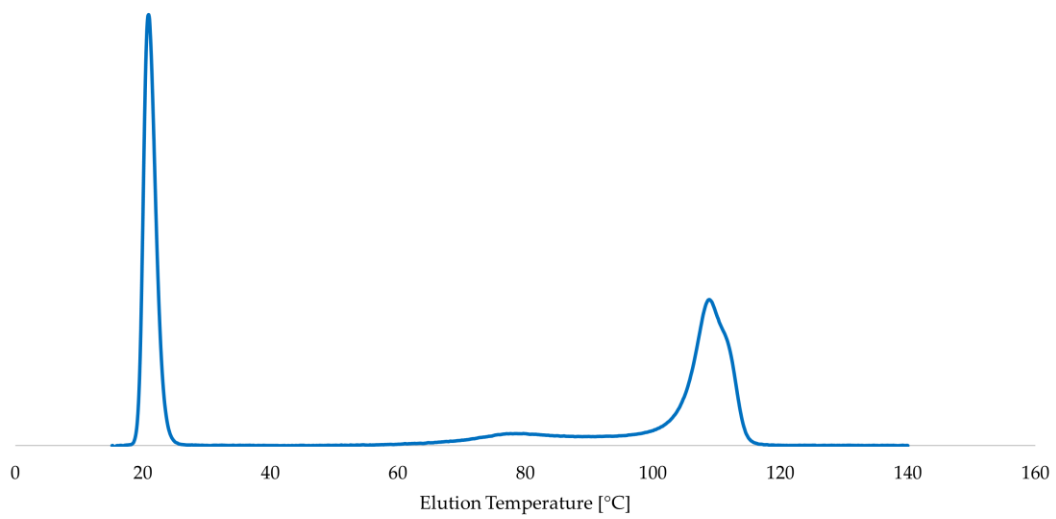

Figure S2. CEF trace of copolymer sample #1 of **Table S5** (soluble fraction SF = 47 wt%).

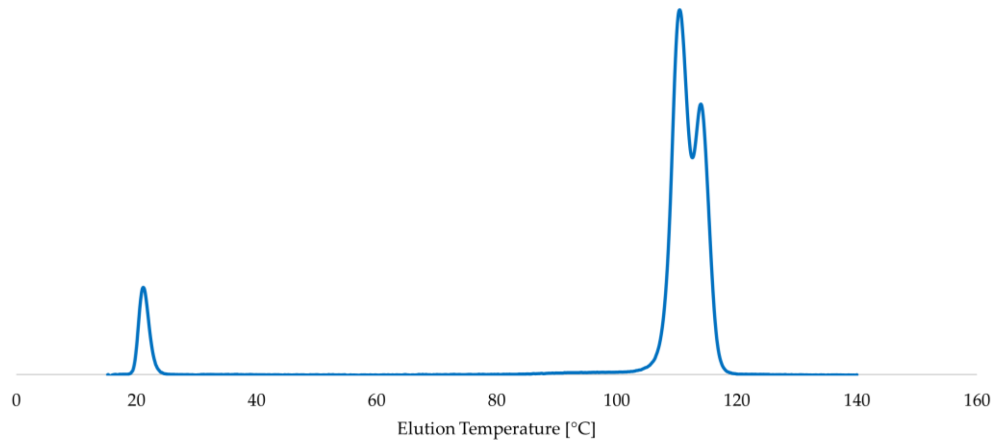

Figure S3. CEF trace of copolymer sample #4 of **Table S5** (soluble fraction SF = 8.3 wt%).

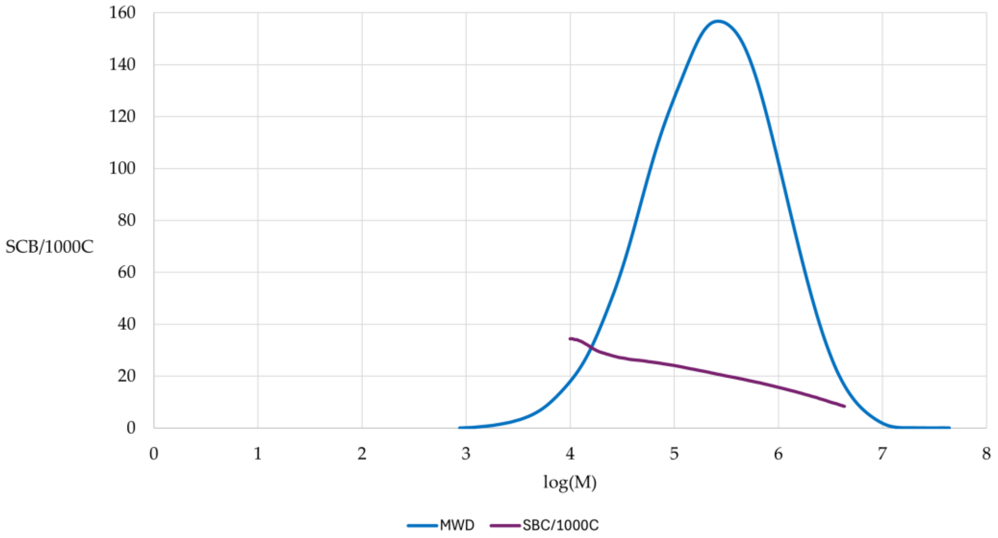

Figure S4. GPC/IR trace of copolymer sample #1 of **Table S5**.

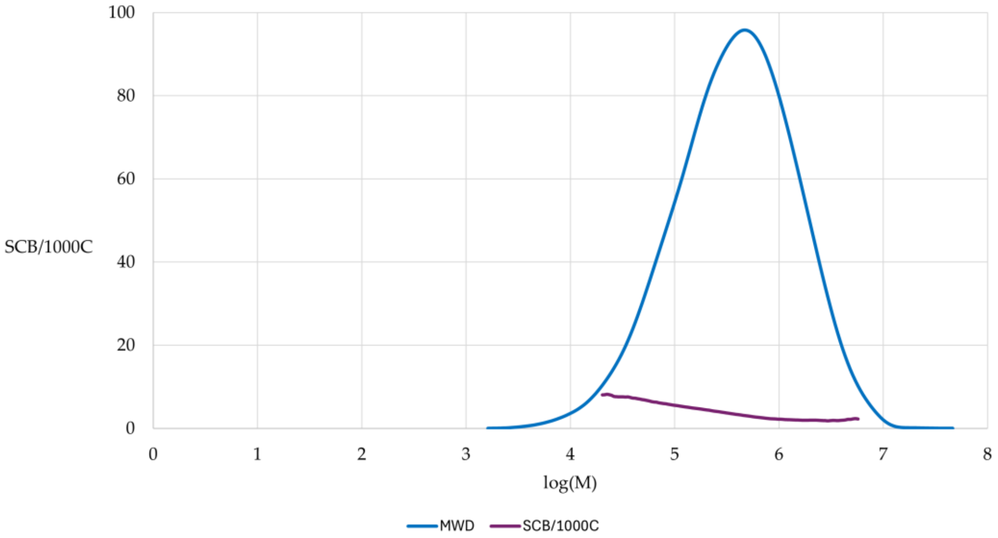

Figure S5. GPC/IR trace of copolymer sample #4 of **Table S5**.

4. Liquid saturation experiments with ethene

Reaction phase saturation experiments with ethene were carried out in the PPR to measure ethene concentration in the different reaction phases of this study. The experimental protocol, aiming to replicate the conditions of real copolymerization experiments (without the catalyst system) is described in the following.

The PPR stir tops were removed, glass vials containing 6.0 mL of the desired liquid phase were positioned in the cells, PEEK paddles were fitted to the stirrers, and the stir tops were then placed back. The modules were heated to 60°C, and after 10 min to allow for stabilization they were pressurized stepwise with ethene up to

a pressure of 65 psi, recording ethene uptake during the process. From the value of ethene uptake at saturation, the concentration of ethene in the liquid phase was calculated assuming ideal behavior in the gas phase at equilibrium. Representative results are shown in **Table S6**.

Table S6. Results of saturation experiments of pure solvents (toluene, 1,2-difluorobenzene, *n*-heptane and decane) with ethene at 60°C in the PPR.

Solvent	Final uptake (psi)	n_E (mol)	[E] (mol/L)
Toluene	65.1	0.00180	0.300
1,2-difluorobenzene	69.2	0.00191	0.318
n-Heptane	72.5	0.00200	0.333
Decane	58.6	0.00162	0.270

The estimated value of [E] in *n*-heptane is in very good agreement with that calculated using the semi-empirical equation of Kissin and Antoine.[10] Similar experiments carried out with liquid phases consisting of toluene/1-hexene solutions yielded identical results within the experimental error.

5. References

1. Ehm, C.; Vittoria, A.; Goryunov, G.P.; Izmer, V.V.; Kononovich, D.S.; Samsonov, O.V.; Budzelaar, P.H.M.; Voskoboynikov, A.Z.; Busico, V.; Uborsky, D.V.; Cipullo, R. On the Limits of Tuning Comonomer Affinity of 'Spaleck-Type' *ansa*-Zirconocenes in Ethene/1-Hexene Copolymerization: A High-Throughput Experimentation/QSAR Approach. *Dalton Trans.* **2020**, *49*, 10162–10172, doi:10.1039/D0DT01967D.

2. Ehm, C.; Mingione, A.; Vittoria, A.; Zaccaria, F.; Cipullo, R.; Busico, V. High-Throughput Experimentation in Olefin Polymerization Catalysis: Facing the Challenges of Miniaturization. *Ind. Eng. Chem. Res.* **2020**, *59*, 13940–13947, doi:10.1021/acs.iecr.0c02549.

3. Busico, V.; Pellecchia, R.; Cutillo, F.; Cipullo, R. High-Throughput Screening in Olefin-Polymerization Catalysis: From Serendipitous Discovery Towards Rational Understanding. *Macromol. Rapid Commun.* **2009**, *30*, 1697–1708, doi:10.1002/MARC.200900246.

4. Taniike, T.; Cannavacciuolo, F.D.; Khoshsefat, M.; De Canditiis, D.; Antinucci, G.; Chammingkwan, P.; Cipullo, R.; Busico, V. End-to-End High-Throughput Approach for Data-Driven Internal Donor Development in Heterogeneous Ziegler-Natta Propylene Polymerization. *ACS Catal.* **2024**, 7589–7599, doi:10.1021/ACSCATAL.4C01601.

5. Ehm, C.; Vittoria, A.; Goryunov, G.P.; Izmer, V.V.; Kononovich, D.S.; Samsonov, O.V.; Di Girolamo, R.; Budzelaar, P.H.M.; Voskoboynikov, A.Z.; Busico, V.; Uborsky, D.V.; Cipullo, R. An Integrated High Throughput Experimentation/Predictive QSAR Modeling Approach to *ansa*-Zirconocene Catalysts for Isotactic Polypropylene. *Polymers* **2020**, *12*, 1005, doi:10.3390/polym12051005.

6. Randall, J.C. A REVIEW OF HIGH RESOLUTION LIQUID ^{13}CARBON NUCLEAR MAGNETIC RESONANCE CHARACTERIZATIONS OF ETHYLENE-BASED POLYMERS. *J. Macromol. Sci. C Polym. Rev.* **1989**, *29*, 201–317, doi:10.1080/07366578908055172.

7. Galland, G.B.; Quijada, P.; Mauler, R.S.; De Menezes, S.C. Determination of Reactivity Ratios for Ethylene/α-Olefin Copolymerization Catalysed by the $C_2H_4[Ind]_2ZrCl_2$/Methylaluminoxane System. *Macromol. Rapid Commun.* **1996**, *17*, 607–613, doi:10.1002/MARC.1996.030170901.

8. Peña, B.; Delgado, J.A.; Pérez, E.; Bello, A. Monomer Sequence Distributions in the Copolymerization of Ethylene and 1-Decene. *Macromol. Chem. Phys.* **1994**, *195*, 2457–2467, doi:10.1002/MACP.1994.021950714.

9. Kakugo, M.; Naito, Y.; Mizunuma, K.; Miyatake, T. ^{13}C NMR Determination of Monomer Sequence Distribution in Ethylene-Propylene Copolymers Prepared with δ-$TiCl_3$-$Al(C_2H_5)_2Cl$. *Macromolecules* **1982**, *15*, 1150–1152, doi:10.1021/MA00232A037.

10. Kissin, Y.V. *Isospecific Polymerization of Olefins*; Springer New York: New York, NY, **1985**; ISBN 978-1-4612-9556-3.
